# Biologically Inspired and Rehabilitation Robotics

**DOI:** 10.1155/2019/2428707

**Published:** 2019-03-13

**Authors:** Liwei Shi, Yong Yu, Nan Xiao, Dongming Gan

**Affiliations:** ^1^The Institute of Advanced Biomedical Engineering System, School of Life Science, Beijing Institute of Technology, No. 5, Zhongguancun South Street, Haidian District, 100081 Beijing, China; ^2^Kagoshima University, Kagoshima, Japan; ^3^Khalifa University Center for Autonomous Robotics Systems and Department of Mechanical Engineering, Khalifa University of Science and Technology, PO Box, 127788 Abu Dhabi, UAE

Intelligent robots will soon be ready to serve in our home, hospital, office, and outdoors. One key approach to the development of such intelligent and autonomous robots draws inspiration from behavior demonstration of biological systems. In fact, using this approach, a number of new application areas have recently received significant interests in the robotics community, including rehabilitation robots, service robots, medical robots, and entertainment robots. It is clear that bioinspired methods are becoming increasingly important in the face of the complexity of today's demanding applications. Biological inspiration in robotics is leading to complex structures with sensory-motor coordination, in which learning often plays an important role in achieving adaptation. In addition, rehabilitation robotics has produced exciting new ideas and novel human assistive devices in the growing field of biomedical robotics. The science and technology of rehabilitation robotics will progress through the collaboration among robotic researchers, medical doctors, and patients.

This special issue focuses on the theoretical and technological challenges of evolutionary transformation from biological systems to intelligent robots. There were 33 submissions received, and 18 original research papers were finally accepted in this special issue after formal peer reviews. The accepted papers can be further classified into three related topics, namely, exoskeleton systems for human assisting and rehabilitation, bioinspired manipulator design for fine manipulation and surgery, and bioinspired sensing system development for human-centered applications.

Among the exoskeleton works, a big focus was on the lower limb applications. A. Yatsugi et al. studied the feasibility of neurorehabilitation using a hybrid assistive limb for patients who underwent spine surgery. Treatment indices were introduced, and real patient subject experiments were conducted to prove that neurorehabilitation therapy using lower limb exoskeleton is feasible following spine surgery. For the similar application, Q. Chen et al. proposed a novel gait planning approach, which is aimed at providing a reliable and balance gait during walking assistance. The exoskeleton and patient were modeled together as a linear inverted pendulum (LIP), and the patients' intention was obtained through an orbital energy diagram. Experimental results demonstrated the effectiveness of the proposed new method. Y. Liu et al. developed a wearable powered foot orthosis with metatarsophalangeal joint which is considered a critical component in human locomotion. The experimental results also suggested that the designed system could offer promise in certain rehabilitation applications and clinical treatment. A soft robotic suit was also developed by S. Jin et al. to assist hip flexion for energy-efficient walking of elderly persons in daily life activities, on metabolic cost reduction in the long-distance level and inclined walking. Experiment results show that, for a 79-year-old healthy male subject, the robotic suit significantly reduced metabolic cost in the condition of the robotic suit worn and powered on compared with the condition of worn but powered off. To assist customized gait planning for stable motion in variable terrains in lower limb exoskeleton-based rehabilitation walking, C. Yue et al. developed a novel wearable sensing system employing 7 force sensors as a sensing matrix to achieve high accuracy of ground reaction force detection. By fusing force and angular velocity data, four typical terrain features are able to be recognized successfully, and the recognition rate can reach up to 93%. For the upper limb rehabilitation, W. Wei et al. developed a soft arm exoskeleton-based Bowden cable-driven system. The movement of the shoulder skeletal system through a mathematical model based on the Bowden cable transmission was explored, and the experimental results show that man-machine interaction force can be reduced when the number of bearing force points is increased and the bearing force point is away from the elbow. To fully understand the human shoulder mechanism to rehabilitation exoskeleton design, a new skeleton model and the motion rhythm analysis for human shoulder complex were proposed by S. Zhibin et al. Experimental data were analyzed and proved the proposed model effectiveness. One of the big topics in exoskeleton research is the human-robot interfacing. EMG has been widely used, and B. Gao et al. studied the real-time evaluation of the sEMG signal processing in an upper arm exoskeleton system for rehabilitation. The experimental results showed that the recognition accuracy of sEMG was 94%, and the average delay time was 300 ms, which met the requirements in the real rehabilitation process.

Biology systems have showed good references and inspiration for engineers to develop new manipulation and sensing systems. A critical review was conducted by H. Al-Shuka et al. with focus on active impedance control of bioinspired motion robotic manipulators. Different strategies were summarized and compared for future reference of new system control development. By learning from human anatomy and the muscle control system, a muscle coordination control for an asymmetrically antagonistic-driven musculoskeletal robot using attractor selection was developed by S. Ide and A. Nishikawa. A virtual antagonistic muscle structure was revealed in the experiments to show the effective control method. By fully exploring the human elbow joint complex, kinematic decoupling analysis was conducted, and a new biomimetic robotic elbow joint was designed by B. Cui et al. Detailed kinematics and design were explained followed by simulation tests. Similarly, a full 7-DOF prosthetic arm was built for shoulder disarticulation with the objective of low cost. A 3D printed method was recommended with the final prototype weighing 1350 g, and assessment was conducted to show the effectiveness in daily living activities. Other than prosthesis, a bioinspired catheter system was also developed by Y. Jiang et al. for endovascular treatment with force feedback. An initial clinical trial was conducted and showed the feasibility of the developed system. Apart from a human, a soft robotic fish was presented by W. Zhao et al. by using piezoelectric fiber composite (PFC) as flexible actuator based on numerical coupling analysis. On the bioinspired sensing side, a novel micro-photoionization detector for rapid volatile organic compound measurement was developed by Q. Zhou et al. The testing results showed that the ion collection efficiency reached 91% at a bias voltage of 150 V. To target microdistance and force perception, a magnetostrictive bioinspired whisker sensor based on the galfenol composite cantilever beam was developed to realize bidirectional tactile perception. In the experiment, the designed whisker, compared with a traditional unimorph whisker, displayed an output voltage range of -240 to 240 mV, the distance was 0-22 mm, with the microforce sensing range of 9.8-2,744 mN, the average distance of 10.90 mm/mV, and the force sensitivity of 11.4 mN/mV. The experimental results show that the proposed whisker sensor can realize the bidirection tactile perception in one-dimensional space. The automatic measurement, especially for products with complex shapes, has always been one of the most important application areas of robots. Aiming at the challenge of measuring residual stress under the curved surface, Q. Pan et al. proposed a robotic system for the residual stress ultrasonic measuring based on combining industrial robot technology with residual stress ultrasonic nondestructive measuring technology. Irregular vibration of the vocal cords corresponding to a variety of voice disorders can be observed with electronic laryngoscope to assist diagnosing vocal cord disease. However, laryngoscopy examination is invasive, and the outcomes are relatively subjective. Acoustic analysis can complement and in some cases replace the other invasive methods, which are based on direct vocal fold observation. Based on this, X. Zhang et al. designed a pathological voice source analysis system by integrating nonlinear dynamics with an optimized asymmetric two-mass model to explore nonlinear characteristics of vocal cord vibration. Changes in acoustic parameters, such as fundamental frequency, caused by distinct subglottal pressure and varying degrees of vocal cord paralysis are analyzed. Experimental results validate the applicability of the proposed model to reproduce vocal cord vibration with high accuracy and show that a paralyzed vocal cord increases the model coupling stiffness.

Therefore, this special issue presents the most recent advances in modeling, design, analysis, implementation, and therapeutic testing of the human assistive rehabilitation robotic exoskeletons, bioinspired prosthesis, manipulators, and sensing systems. We hope the knowledge and information will be good references and basis for further development in those fields for human-centered science and technology.

